# The Canine Oral Microbiome

**DOI:** 10.1371/journal.pone.0036067

**Published:** 2012-04-27

**Authors:** Floyd E. Dewhirst, Erin A. Klein, Emily C. Thompson, Jessica M. Blanton, Tsute Chen, Lisa Milella, Catherine M. F. Buckley, Ian J. Davis, Marie-Lousie Bennett, Zoe V. Marshall-Jones

**Affiliations:** 1 Department of Molecular Genetics, The Forsyth Institute, Cambridge, Massachusetts, United States of America; 2 Department of Oral Medicine, Infection and Immunity, Harvard School of Dental Medicine, Boston, Massachusetts, United States of America; 3 The Veterinary Dental Surgery, Byfleet, United Kingdom; 4 WALTHAM Centre for Pet Nutrition, Melton Mowbray, United Kingdom; 5 Mars Pet Care Europe, Birstall, United Kingdom; Institute for Genome Sciences, University of Maryland School of Medicine, United States of America

## Abstract

Determining the bacterial composition of the canine oral microbiome is of interest for two primary reasons. First, while the human oral microbiome has been well studied using molecular techniques, the oral microbiomes of other mammals have not been studied in equal depth using culture independent methods. This study allows a comparison of the number of bacterial taxa, based on 16S rRNA-gene sequence comparison, shared between humans and dogs, two divergent mammalian species. Second, canine oral bacteria are of interest to veterinary and human medical communities for understanding their roles in health and infectious diseases. The bacteria involved are mostly unnamed and not linked by 16S rRNA-gene sequence identity to a taxonomic scheme. This manuscript describes the analysis of 5,958 16S rRNA-gene sequences from 65 clone libraries. Full length 16S rRNA reference sequences have been obtained for 353 canine bacterial taxa, which were placed in 14 bacterial phyla, 23 classes, 37 orders, 66 families, and 148 genera. Eighty percent of the taxa are currently unnamed. The bacterial taxa identified in dogs are markedly different from those of humans with only 16.4% of oral taxa are shared between dogs and humans based on a 98.5% 16S rRNA sequence similarity cutoff. This indicates that there is a large divergence in the bacteria comprising the oral microbiomes of divergent mammalian species. The historic practice of identifying animal associated bacteria based on phenotypic similarities to human bacteria is generally invalid. This report describes the diversity of the canine oral microbiome and provides a provisional 16S rRNA based taxonomic scheme for naming and identifying unnamed canine bacterial taxa.

## Introduction

Bacteria of the oral cavity have been studied with great interest since Anton van Leeuwenhoek first examined the plaque between his teeth with his crude microscope in 1683 [1]. Using cultivable methods, approximately 300 species from the human oral cavity have been isolated, characterized and formally named. Studies of the oral microbiota of other vertebrates have been less extensive. Unfortunately, bacteria from non-human sources were often misidentified and misclassified based on phenotypic similarity to human microorganisms. With the advent of molecular identification methods, primarily based on 16S rRNA sequence analysis, it has become apparent that bacteria from different vertebrate hosts are frequently unique, despite similar biochemical and other phenotypic traits. While molecular methods have been valuable in clarifying the identification and taxonomy of isolates, the greatest strength of these methods is in the identification of the majority of organisms which are currently uncultivated. Studies with molecular methods have demonstrated that the bacterial diversity in most environments is severely underestimated in surveys with cultivation-based methods [2], [3].

While the human oral microbiome has been surveyed using culture-independent methods [4], the canine oral microbiome has not. Previous canine studies were based primarily on culture-dependant methods and sometimes sought to identify species commonly found in human plaque [5], [6], [7], [8].

The primary purpose of this study was to identify major species of bacteria present in canine oral microbiome through an examination of subgingival plaque using culture-independent methods. This study reports on the analysis of 5,958 16S rRNA sequences from 65 clone libraries and provides 416 full 16S rRNA reference sequences (>1500 base) for the 353 taxa identified. As the vast majority of these taxa are not formally named, a provisional taxonomic scheme is presented based on assigning each taxon to the closest genus or higher taxa, and assigning it a unique Canine Oral Taxon number.

## Materials and Methods

### Ethics Statement

Dogs were recruited in the UK from a kenneled population and from client owned dogs presented at a specialist veterinary clinic; informed client consent was obtained. Two studies were performed as follows: subgingival plaque was collected from 20 dogs in the first study (10 of which were from a kenneled population) and from 31 dogs in the second. The studies were approved by the WALTHAM Centre for Pet Nutrition ethical review committee, and run under licensed authority in accordance with the UK Animals (Scientific Procedures) Act 1986.

### Plaque collection and DNA isolation

Animals were sampled under anesthesia. Each dog was given a premedication of 0.02 mg/kg acepromazine (ACP 2 mg/ml) and 0.02 mg/kg buprenorphine (Vetergesic 0.3 mg/ml) intramuscularly, then induced with 0.4 mg/kg propofol (Rapinovet 10 mg/ml) given intravenously, and maintained on 2% inhalational isoflurane. Initially supra-gingival and gingival margin plaque and calculus were removed using a Gracey curette to prevent contamination of the sub-gingival sample. A periodontal probe was then inserted under the gingival margin and swept along the tooth surface. Plaque from at least eight teeth was pooled. The resulting subgingival plaque pool from each dog was suspended in a 350 µl solution of 50 mM Tris (pH 7.6), 1 mM EDTA (pH 8.0) and 0.5% Tween 20 and was immediately stored at −20°C prior to DNA extraction. DNA extraction was performed using the DNeasy Tissue Kit (Qiagen, Valencia, California) following the manufacturer's instructions for the isolation of genomic DNA from Gram-positive bacteria (which also works well for Gram-negative bacteria). For the second study DNA extraction was performed using the Masterpure Gram Positive DNA Purification Kit (Epicentre, USA), according to the manufacturer's instructions with an additional overnight lysis as follows. Plaque samples were centrifuged at 5000× g for 10 minutes and the cell pellet resuspended in 150 µl of TE buffer (10 mM Tris-Cl and 0.5 mM EDTA, pH 9.0). Following vortexing, 1 µl Ready-Lyse Lysozyme (Epicentre, UK) was added and the lysis mix incubated overnight at 37°C for 18 hrs. Following the extraction, DNA was resuspended in TE buffer.

### DNA amplification

DNA samples purified from subgingival plaque of 20 dogs in study 1 were individually amplified with “universal" primers F24/Y36 (9-29F/1525-1541R) to construct 20 libraries. The sequences of primers are given in Table S1 in the supplemental materials. Purified DNA from the 10 of the 20 dogs was also combined into 4 pools (each pool from 2 or 3 dogs), and each pool was amplified individually with “Bacteroidetes-selective", F24/F01 or “Spirochaetes*-*selective", F24/M98, primers to give eight additional libraries. In study 2, DNA samples purified from subgingival plaque of 31 dogs were individually amplified with “universal" primers F24+AD35/C72 (9-27F [YM+B]/1492-1509R) to construct 31 libraries. The forward primer was a combination of 4 parts of the 4-fold degenerate 9–27 “YM" primer F24 and one part *Bifidobacteriales* primer AD35 (modified from Frank et al. [9], to give a 5-fold degenerate primer mix for enhanced phylogenetic coverage. Equal amount of DNA from 3 sets of ten to eleven dogs were pooled to give 3 DNA super-pools. The three super-pools were amplified individually with “Bacteroidetes-selective", F24/F01 and “Spirochaetes*-*selective", F24/M98, primers to give six additional libraries.

PCR was performed in thin-walled tubes with a Perkin-Elmer 9700 Thermocycler. One µl of the purified DNA template was added to a reaction mixture (50 µl final volume) containing 20 ρmole of each primer, 40 nmole of dNTPs, 2.5 units of Platinum Taq polymerase (Invitrogen, Carlsbad, CA) in 10× PCR buffer (200 mM Tris-HCl pH 8.4, 500 mM KCl). In a hot start protocol, samples were preheated at 94°C for 4 min followed by amplification using the following conditions: denaturation at 94°C for 45 s, annealing at 60°C for 45 s, and elongation at 72°C for 1.5 min with an additional 1 s for each cycle. A total of 30 cycles were performed and then followed by a final elongation step at 72°C for 15 min. The size and amount of each amplicon was examined by electrophoresis in a 1% agarose gel. DNA was stained with SYBR Safe DNA gel stain (Invitrogen, Carlsbad, CA) and visualized under UV light. After checking that a strong amplicon of the correct size was produced, a second preparative gel was run and the full length amplicon band was cut out and purified using a Qiagen Gel Extraction kit (Qiagen, Valencia, CA).

### Cloning and Library Screening procedures

Size-purified PCR amplified DNA was cloned using a TOPO TA Cloning Kit as previously described [4]. Approximately 90 colonies were picked for each library. Clones were amplified using M13 forward and reverse primers and amplicon purified as previously described [4].

### 16S rRNA Sequencing

Purified DNA was sequenced using an ABI prism cycle-sequencing kit (BigDye® Terminator Cycle Sequencing kit) on an ABI 3100 Genetic Analyser (Applied Biosystems, Foster City, CA). The sequencing primers, Table S1 in supplementary materials, were used in a quarter-dye chemistry following the manufacturer's instructions.

### 16S rRNA data analysis

Approximately 500 bases of sequence were determined using primer Y31 (519–533R) to allow preliminary identification of clones. If the clone sequence appeared novel (differing by more than 7 bases from previously identified canine oral reference sequences), a full sequence of approximately 1,500 bases was obtained using 6 to 8 sequencing primers for full double strand coverage (Table S1). The sequencing primers used over the course of the two studies evolved. Primers in Table S1 which failed to produce readable sequence for multiple taxa due to mismatches are labeled “limited" and were not used in subsequent studies. Primers which proved successful empirically and by alignment with human and canine oral reference sequences are labeled “general". Full 16S rRNA sequences were assembled from the ABI electropherogram files using Sequencher (Gene Codes Corporation, Ann Arbor, Michigan). Programs for data entry, editing, sequence alignment, secondary structure comparison, similarity matrix generation, and phylogenetic tree construction were written by F.E. Dewhirst [10]. Consensus neighbor-joining trees [11] were constructed from our aligned sequences using MEGA 4 [12]. The similarity matrices were corrected for multiple base changes at single positions by the method of Jukes and Cantor [13]. Comparisons with missing data were eliminated pairwise. The consensus trees were based on 1,000 bootstrap resamplings.

Sequences were checked for the possibility of being chimeric using a custom program [4] which checked the phylogenetic distance between the best BLAST match of the ends of each sequence with the canine reference set excluding self matches. Sequences whose ends diverged >5% were examined using Mallard [14] and heuristically for sequence consistency with phylogenetic neighbors in our overall sequence alignment sorted phylogenetically.

### Nucleotide Sequences

**Figure 1:**
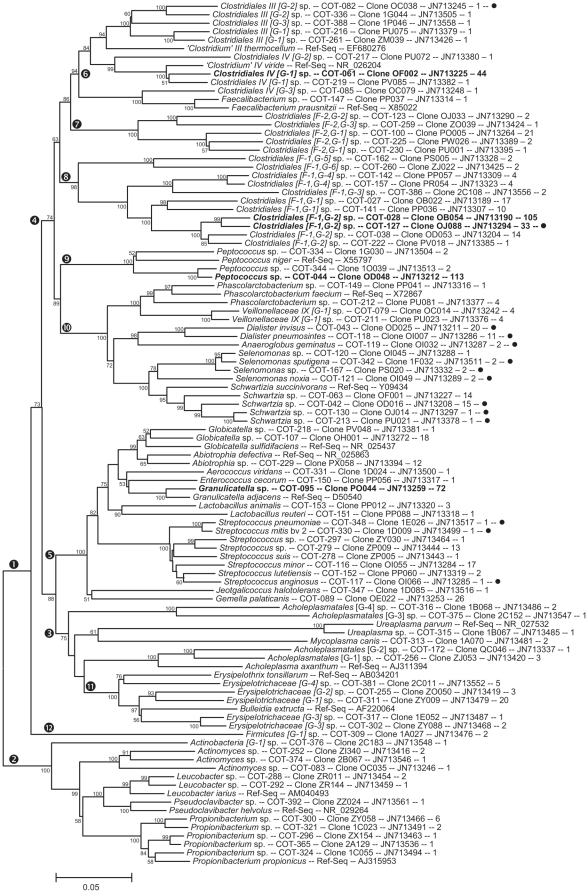
Consensus neighbor-joining tree for canine oral tax in phyla Actinobacteria, Firmicutes and Tenericutes. The name of each taxon is followed by Canine Oral Taxon number, reference clone designation, GenBank accession number, and the number of clones out of 5958 that were identified as this taxon. Taxa marked with a filled circle are also found in the human oral cavity. Taxa for which there were 30 or more clones are shown in bold. The tree was constructed with MEGA 4 using the Jukes and Cantor correction neighbor-joining distance matrix. Comparisons with missing data were eliminated pairwise. The numbers to the left of the branches indicate the percent of time the clade was recovered out of 1,000 bootstrap resamplings. Only bootstrap percentages greater than 50 are shown. Roman numerals following a genus name indicate Collins' *Clostridia* cluster numbers [17] The scale bar shows 5% sequence divergence. The encircled numbers mark clades discussed in the text.

**Figure 2:**
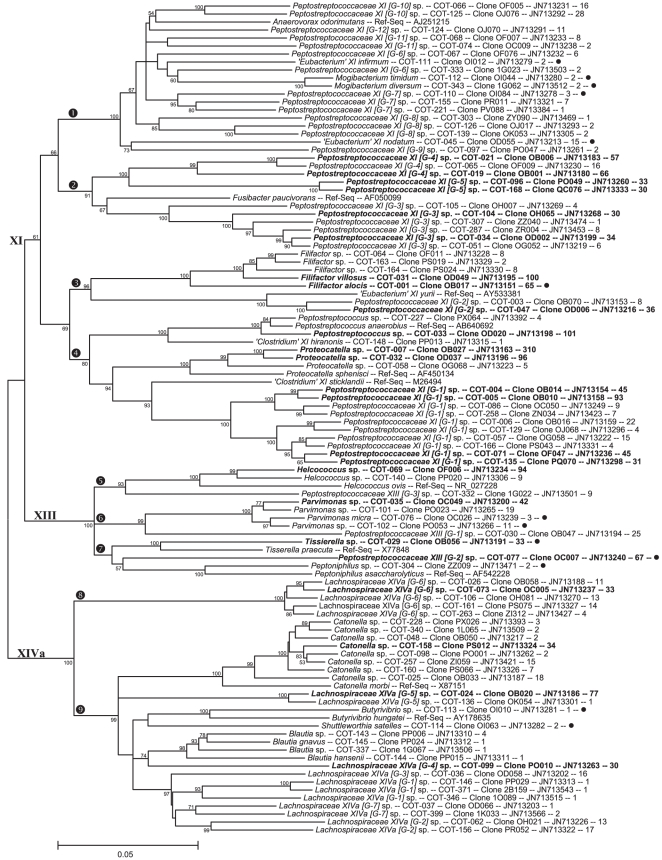
Consensus neighbor-joining tree for class *Clostridia*, families *Peptostrepto-coccaceae* and *Lachnospiraceae*. Labeling and methods used are as described in Fig. 1.

**Figure 3:**
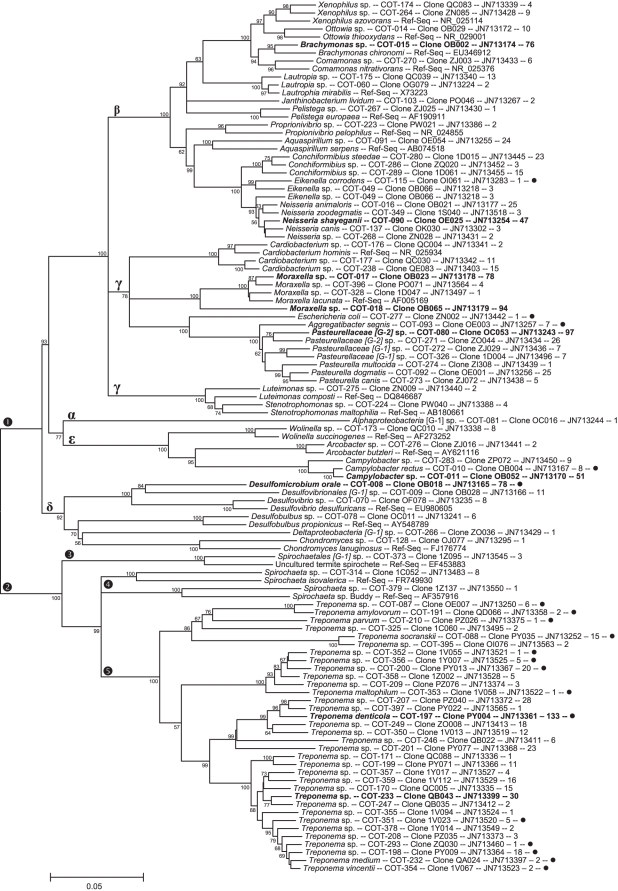
Consensus neighbor-joining tree for phyla Proteobacteria and Spirochaetes. Labeling and methods used are as described in Fig. 1. The Greek letters mark the respective Proteobacteria classes.

**Figure 4:**
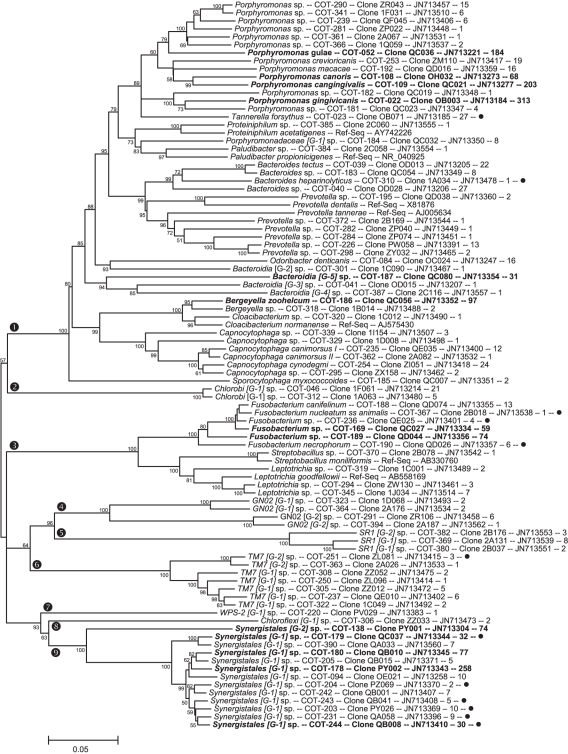
Consensus neighbor-joining tree for phyla Bacteroidetes, Fusobacteria, Chlorobi, Chloroflexi, Synergistetes and candidate divisions TM7, SR1, GN02 and WPS-2. Labeling and methods used are as described in Fig. 1.

The full 16S rRNA sequences for 416 clones representing 353 canine oral taxa were deposited in GenBank and received accession numbers JN713151–JN713566. The accession numbers are also included for each phylotype in Figs. 1, 2, 3, and 4. The partial 16S RNA sequences (the 5′-end ∼500 bases) of 5,959 clones were deposited in GenBank as JQ294075–JQ300033.

## Results and Discussion

Oral samples for 16S rDNA clone library construction came from a wide variety of dog breeds. The breed and age of each dog for each library is given in Table S2 of supplementary materials. The breeds examined include large (Saint Bernard) and small (Papillion) breeds, and those with long (Australian Collie) and short (Shih Tzu) snouts and ranged in age from 3 to 8 years old. While the breeds examined in this study are originally from geographically diverse locations, the dogs sampled are from a limited area of the United Kingdom. Thus, future studies employing samples from dogs living in different countries could well find additional canine microbial diversity. Because the 51 dogs examined came from 25 breeds, there was no attempt to compare microbiomes between breeds as the number of dogs/breed were too low.

### Cloning studies

A total of 6,025 clones were examined from 65 libraries of approximately 90 clones per library. Sixty-seven clones which had sequences shorter than 350 trimmed bases or which were found to be chimeric were excluded for a total of 5,958 validated clones used for analyses. The validated clones from the first cloning library were initially grouped into provisional phylotypes based on their 500 base partial sequences. A full sequence was then determined for a representative of each phylotype. The phylotypes were given arbitrary Canine Oral Taxon numbers (COT-001 through COT-399) in the order they were identified and the full length sequences used as a reference set against which subsequent clones were examined by BLASTN analysis. In this study, a phylotype or COT is defined as a set of one or more 16S rRNA sequences with greater than 98.5% full sequence similarity (23 or fewer base differences for a 1530 base sequence). This phylotype definition was chosen because the 16S rRNA sequence divergence for most strains of named oral species examined is less than 1.5% and inter-species divergence is usually greater than 1.5%. As subsequent clone libraries were screened, any clone with a partial 500-base sequence not matching a reference set sequence by at least 98% (7 base mismatches) was fully sequenced and added as a new reference sequence and given a COT number. Thus all 5,958 partial clone sequences match a reference sequence at a similarity of greater than 98%. Some taxa have two or more reference sequences because members of a taxon can differ by up to 23 base differences and appear <98% similar in their first 500 bases. A total of 353 phylotypes were identified. Seventy of these phylotypes (19.8%) were identified as named species based on greater than 98.5% sequence similarity to a type strains in BLASTN searches of GenBank [15] and Greengenes [16]. The remaining 284 phylotypes (80.2%) represent currently unnamed taxa. As this study made no attempt to cultivate members of the canine microbiome, we are not in a position to address what percent of the unnamed taxa are cultivable or as yet uncultivated as has been done for human taxa [4].

### Taxonomy

Each canine taxon was placed in a phylum or candidate division based initially on BLASTN results against the Human Oral Microbiome Database (HOMD) [4], GenBank databases Reference RNA sequences (refseq_rna) and RNA and Nucleotide collection (nr/nt) [15], and using tools at Greengenes [16]. The Greengenes site was particularly useful for classifying and placing sequences from the rare phyla or candidate divisions Chlorobi, Chloroflexi, GN02 and WPS-2. The 16S rRNA sequences of all canine taxa were placed in an aligned database (hand-aligned based on secondary structure) and analyzed extensively by tree construction anchored to named reference sequences. As was previously done for the human oral microbiome [4], a provisional six level taxonomy was created consistent with the 16S rRNA tree structure. The full taxonomy is presented in Table S3 in supplementary materials. The 353 canine bacterial phylotypes were placed in 14 bacterial Phyla, 23 Classes, 37 Orders, 66 Families, and 148 Genera. The number of taxa and clones in each phylum or candidate division are shown in Table 1.

**Table 1:** Bacterial phyla identified in canine subgingival plaque.

			Clones			
Phyla	Phylotypes	Universal 1525 R^a^	Universal 1492 R^b^	Selective Spiro^c^	Selective Bact^d^	Total
Firmicutes	162	1,148	1,379	0	213	2,740
Proteobacteria	52	224	569	0	68	861
Bacteroidetes	43	213	516	0	420	1,149
Spirochaetes	37	17	22	366	4	409
Synergistetes	13	1	5	511	9	526
Actinobacteria	12	1	11	0	8	20
Fusobacteria	10	0	112	0	58	170
TM7	7	0	7	0	13	20
Tenericutes	6	0	7	0	3	10
GN02	4	0	5	0	6	11
SR1	3	0	0	0	13	13
Chlorobi	2	1	12	0	13	26
Chloroflexi	1	0	2	0	0	2
WPS-2	1	1	0	0	0	1
Total	353	1,606	2,647	877	828	5,958

a Clones from libraries made using 9–27F (F24) and 1525–1541R (Y36) primers.

b Clones from libraries made using expanded coverage 9–27F (F24/AE35) and 1492–1509R (C72) primers.

c Clones from libraries made using “Spirochaetes-selective" F24/M98 primer pair.

d Clones from libraries made using “Bacteroidetes-selective" F24/F01 primer pair.

Shown in Figs 1, 2, 3, and 4 are consensus neighbor-joining trees based on the aligned full 16S rRNA sequences for the 353 canine taxa. Each taxon header includes name (genus and species), Canine Oral Taxon number (COT), clone designation, GenBank accession number, and number of clones identified for each taxon out of a total of 5,958. The 51 taxa with 30 or more clones are shown in bold as major taxa. Those 58 taxa marked with a filled circle are taxa shared with humans, as defined by the canine reference sequences sharing >98.5% similarity with reference sequences in the Human Oral Microbiome Database by BLASTN comparison (www.homd.org). Where a taxon is <90% similar to a named genus, it is designated using the family, or most specific higher taxa name, [G-1] sp. where “[G-1]" indicates it belongs to a novel genus. Family level grouping in the *Clostridia* (Figs 1 & 2) include the widely recognized classification of Collins *et al.* [17]. Thus, *Clostridium viride* is written *‘Clostridium’ IV viride* to indicate it is not in the genus *Clostridium sensu stricto* but rather is a member of Collins Cluster IV.

### Firmicutes and Tenericutes

The majority of taxa in the Firmicutes are shown in Fig. 1 in the cluster marked by encircled “1". The Firmicutes families *Peptostreptococcaceae* and *Lachnospiraceae* are shown in Fig. 2. The phylum Tenericutes, previously the class *Mollicutes* within the Firmicutes [18], is marked with an encircled “3" in Fig. 1. The Firmicutes class *Erysipelotrichi*, marked with an encircled “11", branches within the “phylum" Tenericutes, demonstrating phylogenetic inconsistencies created by elevating class level branches within the Firmicutes to phylum level. One hundred sixty-two taxa were identified as members of the phylum Firmicutes.

### *Clostridia*

The dominant class within the Firmicutes is *Clostridia*, containing 138 taxa. The *Clostridia* clade is shown in Fig. 1, marked encircled “4", and all taxa in Fig. 2. The cluster of 10 taxa, marked encircled “6" in Fig. 1, fall into unnamed genera in Collins Clusters III and IV, except for one taxa falling in the genus *Faecalibacterium*. Sixteen taxa fall into two family level Clusters with no named members, marked encircled “7" and “8", for novel families F-2 and F-1 respectively. Three taxa fall in the family *Peptococcaceae*, marked encircled “9", related to the human associated species *Peptococcus niger*. The family *Veillonellaceae*, previously *Acidaminococcaceae*, is marked encircled “10". We chose not to follow the suggestion of Marchandin *et. al*. [19], to elevate this family to a class as we believe it is taxonomically unjustified. The *Veillonellaceae* cluster contains members of the genera *Dialister, Anaeroglobus, Phascolarctobacterium, Schwartzia, Selenomonas*, and an unnamed genus. Nine of these taxa are also found in humans. Shown in Fig. 2 are those *Clostridia* taxa falling in Collins Clusters XI, XIII, and XIVa, with the first two clusters constituting the family *Peptostreptococceae* and the last cluster the family *Lachnospiraceae*. These two families contain the majority of the *Clostridia* taxa in both dogs and humans. In Collins Cluster XI, the cluster of taxa marked encircled “1" contains 18 taxa. Most are in 7 unnamed genera which may be unique to dogs. This cluster contains some named taxa shared with humans such as *‘Eubacterium’ XI infirmum, Mogibacterium timidum* and *M. diversum*, and *‘Eubacterium’ XI nodatum*. The cluster marked encircled “2" contains 11 taxa in 3 unnamed genera distantly related to *Fusibacter paucivorans*. The cluster marked encircled “3" contains five *Filifactor* species, including *F. alocis* and *F. villosus*, and two taxa related to human associated species *‘Eubacterium’ XI yurii*. The cluster marked encircled “4" contains 16 taxa in the genus *Peptostreptococcus sensu stricto, Proteocatella*, and an unnamed genus distantly related to *‘Clostridium’ XI sticklandii*. The validly named reference bacterium *Proteocatella sphensci* [20] was initially called *‘Frigovirgula patagoniensis’* in GenBank (AF450134) and the name ‘*Frigovirgula’* unfortunately persists causing minor confusion. Within Collins Cluster XIII, clusters marked encircled “5", “6", & “7", are 11 taxa in the genera *Helcococcus, Parvimonas, Tissierella, Peptoniphilus,* and three unnamed genera. Five taxa, including *P. micra,* are shared with humans. Seventeen canine taxa fall in the *Lachnospiraceae* [21], Collins Cluster XIVa, with major subclusters marked encircled “8" and “9". The subclusters contain taxa in the genera *Blautia, Butyrivibrio, Catonella, Shuttleworthia*, as well as 7 unnamed genera. Two taxa, including *S. satelles*, are shared with humans.

### *Bacilli*

The second most dominant class within the Firmicutes is the *Bacilli* with 18 taxa. The *Bacilli* clade is marked with an encircled “5" in Fig. 1. All taxa can be placed in the following genera: *Abiotrophia, Aerococcus, Enterococcus, Gemella, Globicatella, Granulicatella, Jeotgalicoccus, Lactobacillus* and *Streptococcus*. While three streptococcal species are shared with humans, streptococci appear to represent a minor genus in dog. This is not surprising as simple carbohydrates and sugars are not normally a major constituent of the canine diet and canine saliva has a pH of approximately 8.0 (WALTHAM, unpublished data 2011) which may be hostile to members of this aciduric genus.

### *Erysipelotrichi*

Five taxa in this Firmicutes class, marked encircled “11" in Fig. 1, were identified. None were sufficiently close to reference species to place them in the genera *Erysipelothrix* or *Bulleidia*.

### Novel Firmicutes Class

Firmicutes [G-1] sp. COT-309 appears to be a member of a novel deeply branching linage marked encircled “12" in Fig. 1. The closest named species had only 80% sequence similarity, however, a clone from the microbiome of fiber adherent species from rumen fluid was 93% similar (EU844484) supporting this canine taxa as a member of a mammal host associated lineage.

### Tenericutes

Six members of this phylum were identified and are marked encircled “3" in Fig. 1, but excluding the Class *Erysipelotrichi* discussed above. In this tree, the “phylum" does not branch as a monophyletic entity. *Mycoplasma canis* and an *Ureaplasma parvum*-related taxon can be placed in named genera, but four additional taxa fall into unnamed genera.

### Actinobacteria

Twelve Actinobacteria were identified and are marked encircled “2" in Fig. 1. Taxa in the genera *Actinomyces, Leucobacter, Pseudoclavibacter, Propionibacterium*, were identified as well as a deeply branching taxa Actinobacteria *[G-1]* sp. COT-376. None of these canine oral taxa are shared with humans. In study 1 using the standard 9–27F and 1525–1541R primers, only one Actinobacteria clone was recovered. Because the 1525–1541R primer has been reported to discriminate against Actinobacteria [22], we switched to the 1492–1505R primer in hopes of obtaining less biased coverage in our second study. Eleven clones were obtained with the revised “universal" primers and eight additional clones by fortuitous mispriming using the “Bacteroidetes-selective" primer set. It appears that no truly “universal" 16S rRNA primers exist and studies of diversity benefit from the use of multiple primer sets. *Actinomyces* sp. COT-083 fell in the genus *Actinomyces*, and is 97% similar to *Actinomyces coleocanis*, a species isolated from the vagina of a dog [23].

### Proteobacteria

Fifty-two phylotypes were identified from the phylum *Proteobacteria*, and are marked with an encircled “1" in Fig. 3. The five classes are marked with Greek letters. The 22 *Betaproteobacteria* taxa include 11 from the mammalian host associated genera *Neisseria, Eikenella* and *Conchiformibius*. Whether the taxa associated with other genera in the *Betaproteobacteria* are truly part of the endogenous oral microbiome, or are transient common environmental bacteria remains to be determined. The 18 *Gammaproteobacteria* taxa include the host associated genera *Cardiobacterium*, *Moraxella* and species in the families *Pasteurellaceae* and *Enterbacteriaceae*. Taxa in the genera *Luteimonas* and *Stenotrophomonas* may be transient common environmental bacteria. One deeply branching *Alphaproteobacteria* taxon, distantly related to named species (81% similarity), was identified. The five *Epsilonproteobacteria* and six *Deltaproteobacteria* taxa are related to well-known mammalian host associated genera except for *Chondromyces*, which is generally associated with soil or decaying organic matter.

### Spirochaetes

Thirty-seven phylotypes from the phylum Spirochaetes were identified and are marked by encircled “2" in Fig. 3. Thirty-four taxa are members of the genus *Treponema*, marked encircled “5", including the named species *T. amylovorum, T. denticola, T. maltophilum, T. medium, T. parvum, T. socranskii,* and *T. vincentii* which are also found in the human oral cavity. A total of 14 canine *Treponema* spp. are shared with humans. Unlike previous studies of the human oral cavity [4], three taxa outside the genus *Treponema* were identified and marked encircled “3" and “4". *Spirochaeta* sp. COT-379 is most closely related to *Spirochaeta coccoides* (NR_042260; not shown) and *Spirochaeta* sp. Buddy. These two species are not helical cells, typical of spirochetes, but rather have a coccoid morphology. *Spirochaeta* sp. COT-314 is 92% similar to a strain isolated from the marine bristle worm *Alvinella pompejana* (AJ431240; not shown) and *Spirochaeta isovalerica*. *Spirochaetes [G1]* sp. COT-373 is a deeply-branching taxa with 93% similarity to a clone sequence from the termite gut, EF453883. Thus it appears that the diversity of spirochetes in the mammalian oral cavity may be broader than just the genus *Treponema*. The vast majority of the spirochete clones came from the 7 libraries produced using “spirochete-selective" primers (Table 1), which demonstrates the utility of using selective primers.

### Bacteroidetes

Forty-three phylotypes were identified as members of the phylum Bacteroidetes, marked by encircled “1" in Fig. 4. Eleven named genera include: *Porphyromonas, Tannerella, Proteiniphilum, Paludibacter, Bacteroides, Prevotella, Odoribacter, Bergeyella, Cloacibacterium, Capnocytophaga* and *Sporocytophaga*, and 5 unnamed deeply branching genera what are not anchored to named taxa. The use of “Bacteroidetes-selective" primers with DNA from 7 super-pools produced 420 clones in the Bacteroidetes phylum and increased the depth and diversity of taxa identified over that produced from “universal" primers (Table 1).

There are naming issues for a number of species in the Bacteroidetes phylum. *Porphyromonas gingivicanis* and *Porphyromonas crevioricanis* were properly named and validly published by Hirasawa & Takada in 1994 [24]. Unfortunately, no 16S rRNA sequences for the type strains of these species were deposited by anyone for 12 years (see DQ677835 & DQ67736) and for 14 years by the authors (see AB430828 & AB430829). While these sequences were unavailable, Collins *et al*. named *Porphyromonas cansulci* [25] and deposited its 16S rRNA sequence in GenBank as entry X76260. “*Porphyromonas canis*" was invalidly named by Sakamoto & Benno in 1999 as GenBank entry AB034799. From the 16S rRNA sequences, we now know that *P. cansulci* is a synonym for *P. crevioricanis*, and that *“P. canis"* is an invalid synonym for *P. gingivicanis*. *Odoribacter denticanis* was named and validly published by Hardham *et al.* [26], but was challenged by Euzeby in comments in the List of Prokaryotic Names with Standing in Nomenclature (http://www.bacterio.cict.fr/) for not having a type strain available. This appears to be rectified as the type strain is now available from three national collections. This species was also previously referred to as “*Wernerella denticanis*" and “*Porphyromonas denticanis*". *Bacteroides* sp. COT-183 has been called “*Bacteroides denticanum*" by Elliott (see DQ156993) and “*Bacteroides denticanoris*" by Hardham *et al.* (see AY54431) in GenBank and patent filings, but never validly described in any publication.

### Chlorobi

Two phylotypes from the phylum Chlorobi, marked with encircled “2" in Fig. 4, were identified. The original cultivable members of the phylum *Chlorobi*, previously called Green Sulfur Bacteria or *Chlorobia*, are phototropic organisms [27]. Cultivation independent molecular methods have identified members from diverse environments. Recently a non-photosynthetic member of the phylum, *Ignavibacterium album*, has been described [28]. Sequences in GenBank with greater than 84% similarity to canine *Chlorobi* phylotypes COT-046 & COT-312 have been recovered from manure drainage, penguin dropping sediment, hydrothermal worm mucus, and from an anaerobic digester. A sequence with 99% similarity to COT-046 has been recovered from the oral cavity of a cat (unpublished observation), supporting the association of this taxa with the oral cavity of mammals. Nine clones from skin swabs of the volar forearms of four human subjects (based on subject identification number in GenBank entries) have a sequence similarity of 99% to canine *Chlorobi* taxa (for example HM278300 and HM330153 to COT-046). These four human subjects appear to have had the volar surface of their arms licked by dogs prior to sampling as their clone libraries include 23 to 51 canine oral taxa.

### Fusobacteria

Ten taxa from the phylum Fusobacteria, marked encircled “3" in Fig. 4, were identified, including the genera *Fusobacterium, Streptobacillus*, and *Leptotrichia*. The Fusobacteria spp. includes four taxa that overlap the human *F. nucleatum* cluster. *Streptobacillus* sp. COT-370 is closely related to the rat bite fever organism *S. moniliformis*. It was suggested previously that dogs may be colonized with *S. moniliformis* by eating rats [29], but the current study suggests that dogs may be naturally colonized with a distinct, but closely related species. It is notable that clones from this phylum were not present in the 10 libraries made by PCR with standard 9–27F and 1525–1541R primers, but were present (110 clones) in 21 libraries using an extend specificity 9–27F and 1492–1509R primers (see methods and Table S1).

### GN02

Four taxa from the as-yet-uncultured GN02 candidate division, marked encircled “4" in Fig. 4 were identified. GN02 is one of 15 candidate divisions proposed in a study of the Guerrero Negro hypersaline microbial mat [30]. The canine phylotypes were originally placed in this division using BLASTN searches of the Greengenes database. In the past year, related taxa from human mouth and skin have started to appear in GenBank as human microbiome data have been submitted (for example FJ976283 & HM249743).

### SR1

Three taxa from the as-yet-uncultured SR1 candidate division, marked encircled “5" in Fig. 4 were identified. The SR1 division was named for clones identified in a study of sediment with microbial streamers from the Sulphur River in Parkers Cave, Kentucky [31]. The SR1 division was previously part of candidate division OP11, so older references to a closely related taxa from the human oral cavity referred the human taxon as OP11 clone X112 [32]. The human phylotype, now designated SR1 sp. HOT-345, has been identified in multiple clone libraries [4].

### TM7

Seven canine phylotypes were identified as members of the candidate division TM7, which is marked with an encircled “6" in Fig. 4. The phylum TM7 is a major lineage of *Bacteria* with no known pure-culture representatives [33]. TM7 organisms have been recognized in 16S rRNA cloning studies of many habitats, including soils, fresh ground water, seawater, and mammalian clinical samples [33]. They have been recovered from the human oral cavity [4], [32], [34], the human distal esophagus [35], and mouse feces [36].

### WPS-2

The candidate division WPS-2, marked with encircled “7" in Fig. 4, is known from only 39 environmental clones in Greengenes otu_4420, mainly from soils. The WPS-2 division was one of two named for clones identified in a study of Wittenberg polluted soil, Germany [37]. WPS-2 sp. COT-220 is closest to GenBank entry DQ520181, and is the 40^th^ member of this rarely observed candidate division marked encircled “7" in Fig. 4. As this taxon was detected as a single clone, and no related clones have been identified from human or other mammalian sources, it remains to be determined if this taxon is part of the endogenous canine oral microflora, or an environmental transient.

### Chloroflexi

A single phylotype of the Chloroflexi phylum was identified and is marked with encircled “8" in Fig. 4. The Chloroflexi phylum, previously called green non-sulfur bacteria, has many cultivated species [38], and several were named subsequent to the description in Bergey's Manual of Systematic Bacteriology [39]. The canine Chloroflexi sp. COT-306 is 96% similar to human oral taxon Chloroflexi sp. HOT-439 and 86% similarity to named species *Anaerolinea thermophila* [40] in the class *Anaerolineae* [41].

### Synergistetes

The phylum Synergistetes is known mainly from clone sequences, but contains about a dozen cultivated species including *Synergistes jonesii*, a rumen bacterium that degrades toxic pyridinediols [42] and *Pyramidobacter piscolens*, a species from the human oral cavity [43]. Organisms from the Synergistetes phylum have previously been mistakenly included in the phylum *Firmicutes* or placed in the phylum Deferribacteres (a sister phylum of Synergistetes and Flexistipes) [32]. As marked by an encircled “9" in Fig. 4, 13 canine phylotypes were identified. Six canine phylotypes match previously identified human phylotypes at >98.5% similarity [4].

### Primer biases

The number of clones identified in each phylum for libraries generated with two different “universal" primer pairs, a “Spirochaetes-selective" pair, and a “Bacteroidetes-selective" pair are shown in Table S1. A marked difference in the diversity recovered in clone libraries using different initial PCR primers is apparent. In study 1, the commonly used “universal" 9–27 YM forward (F24) and 1525–1541 reverse (Y36) primers produced more than one clone only for the four common phyla Firmicutes, Proteobacteria, Bacteroides, and Spirochaetes. In the second study, using expanded coverage 9–27 forward primers (F24/AD35) [9] and the “universal" 1492–1509 reverse primer (C72), clones from 12 phyla/candidate divisions were recovered. Of particular note is the recovery of Fusobacteria taxa only with the second set of “universal" primers and recovery of significantly more Actinobacteria clones with the second primer set. PCR with the “Spirochaetes-selective" reverse primer M98 (1483–1501) yielded expected results: organisms from the Spirochaetes and Synergistetes phyla. Bacteria in these two taxa have “GG" at position 1484-5 whereas most other bacteria have “CT". The “Bacteroidaetes-selective" reverse primer F01 (1487–1505) selects for organisms with a “CT" at position 1490-1 whereas most non Bacteroidaetes have other bases at these positions. While the F24/F01 primer set yielded mostly clones from the Bacteroidetes phylum, clones for 12 phyla/candidate divisions were recovered. The recovery of Chlorobi clones was expected based on perfect primer sequence match; the recovery of TM7 and SR1, which have a one base mismatch “TT", is also expected; but recovery of other taxa, such as Firmicutes, Proteobacteria and Fusobacteria, is somewhat unexpected as they have 2 base mismatches. While the “Spirochaetes–selective" primers are truly selective, the “Bacteroidetes-selective" primers produced clones from 12 of 14 phyla and appear to be useful in recovering a number of rare phyla/candidate divisions. The recovery of taxa from diverse phyla was clearly aided by using multiple primer sets for PCR of DNA prior to library construction. Because this study used taxa selective primers (as all studies ultimately do) to construct libraries, it is impossible to say anything valid about relative abundance of canine oral species from the abundance of clones recovered.

### Taxa abundance

The rank abundance of clones for each canine oral taxon is presented in Table S4 in supplemental materials. Because a variety of primers with various biases were used for library construction, the clone abundance data reflect only clone numbers found in these libraries and cannot be used to validly infer the underlying population structure. With the caveat noted, the most prevalent taxa, *Porphyromonas gingivicanis* COT-022, constituted 5.3% of the clones. Clones from 28 taxa were recovered at level of greater than one percent. The 89 singleton clones were present as 0.017% of 5,958 clones identified. Of the 50 most common taxa, it is striking that 40, or 80%, are unnamed. The taxon rank abundance profile for this canine study is very similar to that previously found for the human oral microbiome [4]. In the human study of about 35,000 clones, it was estimated that the number of taxa necessary to identify 90%, 95% and 98% of the clones was 259, 423 and 655 taxa respectively. Assuming the canine and human oral cavities contain about equal microbial diversity and similar rank abundance profiles, 353 canine taxa should allow identification of about 93% of clones in a study of similar size. This estimate is approximate, but suggests that 353 taxa capture a significant portion of the microbiome. While the current study provides good initial coverage of the canine oral microbiome, the oral samples examined were limited to the subgingival sites. Further studies sampling other oral habitats such as teeth, tongue, cheek, hard and soft palates, and tonsils will no doubt expand the number of canine taxa to approach the more than 1,000 currently defined for the human oral microbiome [4]. One goal of the current study was to obtain essentially full length 16S rDNA reference sequences, which are required for recognition and placement of previously unrecognized rare taxa members such as those in candidate divisions GN02 and WPS-2. Future studies using next generation sequencing methods will no doubt sequence more deeply, producing tens to hundreds of thousands of short sequences. Studies require tradeoffs between sequence length (full length better for phylogenetic studies), and sequence number (higher numbers better for determining community composition).

Comparison of reference 16S rDNA sequences from the canine oral cavity with those of the human oral cavity reveals that only 16.4% of the taxa are shared by BLASTN analysis at a threshold of 98.5% sequence similarity (see taxa marked with filled circle in Figs. 1, 2, 3, and 4). This indicates that there is a large divergence in the oral microbiomes of divergent mammalian species. Of the 83.6% of taxa that differ, the differences are not only at the species level, but also at genus through phylum levels. It is apparent from the results presented here, however, that the majority of oral bacteria from divergent mammalian species are unique and the practice of naming mammalian (or even more distantly related animal) isolates after the most phenotypically similar species from humans is likely to be shown invalid by using molecular tools.

### The Canine Oral Microbiome

The provisional taxonomic scheme presented in supplementary materials Table S3, and the linked 16S rRNA reference sequences, provide the most comprehensive resource to date for identifying and referencing both the named and the 80% as yet unnamed canine oral taxa. This sequence based identification resource should facilitate future molecular studies of canine health and disease as well as the zoonotic potential of canine oral microbes in human and veterinary infectious diseases. The taxonomic scheme presented here currently includes only those taxa for which clones were identified in this study. It is anticipated future efforts will expand this taxonomy and reference sequence set to include all named canine-associated species, and isolates of novel taxa, for which full length 16S rRNA sequences exist.

### Conclusions

The results of this study provide the groundwork for describing the diversity of taxa present in the canine oral cavity. The provisional scheme of giving each taxon a canine oral taxon number and placing it in a phylogenetic context should facilitate future studies of the canine oral microbiome and its role in canine health and disease. The canine oral microbiome is widely divergent from that of human, hence these results will also help in the interpretation of human microbiome studies where canine oral bacteria appear to be present in large numbers in certain human skin samples and in veterinary and human medical studies where previously unnamed canine taxa are recovered from clinical samples.

## Supporting Information

Table S1 PCR and sequencing primers.
(DOCX)

Table S2 16S rDNA clones libraries.
(XLSX)

Table S3 Canine taxonomy.
(XLS)

Table S4 Rank abundance of clones in canine oral taxa.
(XLS)
