# Correction: The Canine Oral Microbiome

**DOI:** 10.1371/annotation/c2287fc7-c976-4d78-a28f-1d4e024d568f

**Published:** 2012-06-08

**Authors:** Floyd E. Dewhirst, Erin A. Klein, Emily C. Thompson, Jessica M. Blanton, Tsute Chen, Lisa Milella, Catherine M. F. Buckley, Ian J. Davis, Marie-Lousie Bennett, Zoe V. Marshall-Jones

The published funding and competing interests statements were incorrect.

The correct funding statement is: No current external funding exists for this study.

The correct competing interests statement is: We have the following interests. CMFB, IJD, M-LB and ZVM-J were employees of WALTHAM Centre for pet nutrition during the course of this study and M-LB is currently at Mars Pet Care Europe. FED, EAK, ECT, JMB, TS were employees of the Forsyth Institute during the course of this study and LM is from the Veterinary Dental Surgery. FED has consulted for WALTHAM. FED CMFB, M-LB, and ZVM-J are inventors on patent WO2008137541, Dog Plaque Health, filed by WALTHAM. There are no other patents, products in development or other marketed products to declare. This does not alter our adherence to all the PLoS ONE policies on sharing data and materials, as detailed online in the guide for authors.

The publisher apologizes for the error. 

