# Supplementary material for: The Canine Oral Microbiome
Source: PLoS One. 2012 Apr 27;7(4):e36067. doi: 10.1371/journal.pone.0036067 (PMC3338629; doi:10.1371/journal.pone.0036067)
Supplement: Table S1 — PCR and sequencing primers. (DOCX) [file pone.0036067.s001.docx]

Table S1. PCR and sequencing primers used in this study

| Primer | Position^b^ | Orientation | Specificity^c^ | Sequence |
| --- | --- | --- | --- | --- |
| F24 | 9-27 | Forward | General | GAGTTTGATYMTGGCTCAG |
| AD35^a^ | 9-27 | Forward | Bifidobacteriales | GGGTTCGATTCTGGCTCAG |
| AD30 | 342-357 | Forward | General | CTRCGGRAGGCAGCAG |
| AC08 | 519-533 | Forward | General | CAGCAGCCGCGGTMA |
| Z24 | 789-806 | Forward | Limited | TAGATWCCCYGGTAGTCC |
| Y34 | 1100-1114 | Forward | General | YAACGAGCGCAACCC |
| AD29 | 342-357 | Reverse | General | CTGCTGCCTYCCGYAG |
| Y31 | 519-533 | Reverse | General | TKACCGCGGCTGCTG |
| Z25 | 907-926 | Reverse | Limited | CCGTCWATTYMTTTRAGTTT |
| Y33 | 907-926 | Reverse | Limited | CCGTCAATTCCTTTRWGTTT |
| AC09 | 1100-1114 | Reverse | General | GGGTTGCGCTCGTTR |
| Z26 | 1374-1391 | Reverse | Limited | AAGRCCCGRRAACGKATT |
| Z27 | 1389-1404 | Reverse | Limited | GGGCGGTGTGTACAAG |
| M98 | 1483-1501 | Reverse | Spirochaetes | GTTACGACTTCACCCYCCT |
| F01 | 1487-1505 | Reverse | Bacteroidetes | CCTTGTTACGACTTAGCCC |
| C72 | 1492-1509 | Reverse | General | GYTACCTTGTTACGACTT |
| Y36 | 1525-1541 | Reverse | General | GAAGGAGGTGWTCCADCC^d^ |

^a^Primer combined with F24 for more universal forward.

^b^Position using *E. coli* numbering.

^c^Primers selective for specific taxonomic groups are so labeled. Primers labeled “Limited” were found to be of limited used and no longer used or recommended. Primers labeled “General” have been found suitable for use nearly all oral taxa and are recommended.

^d^Primer Y36 has a 5’-G for better addition of an overhanging A on the complementary strand for TA cloning.
